# Childhood Hypercalciuric Hypercalcemia With Elevated Vitamin D and Suppressed Parathyroid Hormone: Long-Term Follow Up

**DOI:** 10.3389/fped.2021.752312

**Published:** 2021-11-10

**Authors:** Evgenia Gurevich, Shelly Levi, Yael Borovitz, Hadas Alfandary, Liat Ganon, Dganit Dinour, Miriam Davidovits

**Affiliations:** ^1^Schneider Children's Medical Center of Israel, Pediatric Nephrology Institute, Petach Tikva, Israel; ^2^Faculty of Health Sciences, Ben Gurion University of the Negev, Beer Sheva, Israel; ^3^Sackler School of Medicine, Tel Aviv University, Tel Aviv, Israel; ^4^Department of Nephrology and Hypertension, The Chaim Sheba Medical Center, Tel-Hashomer, Israel

**Keywords:** hypercalcemia, hypercalciuria, nephrocalcinosis, CYP24A1 mutation, SLC34A mutation

## Abstract

**Purpose:** Hypercalcemia with low parathyroid hormone (PTH) level, hypercalciuria, nephrocalcinosis, or nephrolithiasis, was recently reported as caused by mutations in CYP24A1 and SLC34A genes. These encode for vitamin D-24A-hydroxylase and for the renal phosphate transporters NaPiIIa and NaPiIIc, respectively. We aimed to describe the clinical course of these monogenic disorders in patients with and without found mutations during long-term follow-up.

**Methods:** Ten patients with hypercalcemia, hypercalciuria, elevated 1,25-(OH)_2_D levels and suppressed PTH were followed in our center during 1998–2019. Relevant laboratory and imaging data and results of genetic evaluation were retrieved from medical files.

**Results:** The median age at presentation was 9.5 months (range 1 month−11 years), six were males, and the median follow-up time was 3.8 (1.1–14) years. Mutations in CYP24A1 and SLC34A3 were identified in three and one patients, respectively. Five patients presented with nephrocalcinosis, three with nephrolithiasis, and two had normal renal ultrasound. High blood calcium and 1,25-(OH)_2_D levels at presentation decreased during follow-up [11.1 ± 1 vs. 9.9 ± 0.5 mg/dl (*p* = 0.012), and 307 ± 130 vs. 209 ± 65 pmol/l (*p* = 0.03), respectively]; this paralleled an increase in suppressed PTH levels (5.8 ± 0.9 vs. 11.8 ± 7.3 pg/ml, *p* = 0.2). Substantial improvements in hypercalciuria and renal sonography findings were not observed. Two patients had impaired renal function (eGFR 84–88 ml/min/1/73 m^2^) at the last follow up. Interventions included appropriate diet, citrate supplementation, and thiazides.

**Conclusion:** Despite improvement in hypercalcemia and 1,25-(OH)_2_D levels, not all the patients showed improvements in hypercalciuria and nephrocalcinosis. Deterioration of renal function was also observed. Long-term follow up and intervention to prevent nephrocalcinosis and nephrolithiasis are recommended in these children.

## Introduction

Infantile hypercalcemia, hypercalciuria, nephrocalcinosis, and nephrolithiasis are caused by dysregulation of calcium or phosphate homeostasis. The active metabolite of vitamin D, 1,25-dihydroxivitamin D [1,25-(OH)_2_D], plays an important role in calcium and phosphate metabolism, by increasing their intestinal absorption and calcium reabsorption in the kidney (1). Catabolism of vitamin D includes several steps of hydroxylation catalyzed by mitochondrial enzyme 24-hydroxylase, which is encoded by gene CYP24A1. A defect in this process leads to a common biochemical profile, characterized by elevated 1,25-(OH)2D, absorptive hypercalcemia, hypercalciuria, nephrocalcinosis, or nephrolithiasis; and suppressed PTH.

In 2011, Schlingmann et al. (2) identified loss-of-function mutations in CYP24A1 in children with idiopathic infantile hypercalcemia and the abovementioned biochemical profile. These mutations were shown to markedly reduce the enzyme's catabolic activity.

Phosphate regulation occurs in the kidney, predominantly in the proximal tubule, where up to 80% of filtered phosphate is reabsorbed *via* the phosphate transporters NapiIIa and NapiIIc, encoded by genes SLC34A1 and SLC34A3, respectively. Phosphate transporter Napi2c is expressed also in the intestine, where it is responsible for phosphate absorption. A defect in one of these genes leads to hypercalciuria consequent to primary phosphate loss in the kidney, followed by downregulation of FGF23. The result is increased 1,25-(OH)_2_D activity, hypercalcemia, and PTH suppression, as was described by Schligmann et al. (3). Phosphate depletion in itself also enhances 1,25-(OH)_2_D production (4).

Since the discovery of the mutations described above, an increasing number of case reports have been published (2, 3, 5–9), but the natural history and the appropriate treatment of these conditions are still to be defined. In this study, we describe the follow up of 10 pediatric patients who presented to our referral center with hypercalcemia, nephrocalcinosis, or nephrolithiasis, and with a typical biochemical profile of elevated 1,25-(OH)_2_D and suppressed PTH. They all underwent genetic evaluation for mutations in genes CYP24A1, SLC34A1, and SLC34A3. The aim of this study was to describe the course and prognosis of these conditions in patients with and without found mutations in relevant genes during long-term follow-up.

## Materials and Methods

This is a retrospective cohort study. The study protocol was approved by the local committee on human research. Patients presented to our pediatric nephrology institute during 1998–2019 with characteristic laboratory findings of hypercalcemia or hypercalciuria, suppressed PTH, and elevated 1,25-(OH)_2_D, with or without a sonographic picture of nephrocalcinosis or nephrolithiasis were included. The ethics committee granted a waiver for informed consent.

Clinical, biochemical and imaging data, and treatment approaches were retrieved from medical charts. These entailed laboratory results including serum calcium, phosphate, creatinine, 25 (OH) and 1,25-(OH)_2_D levels; PTH levels; and the urine calcium/creatinine ratio, which were collected every 3 months in the first year after initial diagnosis, every 6 months in the second year, and at yearly intervals thereafter.

PTH was determined using the Elecsys PTH (1–84) immunoassay.

1,25-(OH)_2_ D was assessed with the LIAISON 25 OH Vitamin D Total Assay using CLIA technology for the quantitative determination of hydroxylated vitamin D metabolites. The same methods for laboratory assessment were used during the follow up.

Serum creatinine was measured by the modified Jaffe method until 2009 and by an enzymatic method thereafter. Renal function was assessed by glomerular filtration rate (GFR) (ml/min/1.73 m^2^) estimation using the Schwartz formula: 0.45–0.55 × height (cm)/serum creatinine (mg/dl) up to 2009 and 0.413 × height (cm)/serum creatinine (enzymatic) thereafter (10, 11).

Ultrasound examinations and nephrocalcinosis severity grading were assessed by the same experienced radiologist, according to the grading of nephrocalcinosis (12).

Genetic analysis for all the patients was performed in the molecular laboratory at Sheba Medical Center, Israel, after approval of the local Helsinki committee. Genomic DNA was isolated from peripheral blood cells, applying the ArchivePure DNA Blood Kit (5 PRIME, USA) according to the manufacturer's instructions. The coding sequence and splice-sites of CYP24A1, SLC34A3 and SLC34A1 were amplified by PCR using intronic primers. Intronic mutations were not screened. All the PCR products were sequenced directly (ABI Prism 3100; Applied Biosystems, Foster City, CA).

## Results

### Clinical and Laboratory Data at Presentation

Ten patients, aged 1 month−11 years (median: 9.5 months) at presentation were included. Six patients were males. The median follow-up time was 3.8 (1.1–14) years.

The clinical and laboratory characteristics of each patient at diagnosis and at the last follow up are presented in Tables 1 and 2, respectively.

**Table 1:** Clinical and laboratory data at presentation.

**Patient *N***	**Gender**	**Age**	**Ca blood (mg/dl)**	**P blood (mg/dl)**	**PTH (pg/ml) (*N* 14–53)**	**1,25-D (pmol/l) (*N* 39–160)**	**Ca/Crea urine (mg/mg)**	**Clinical presentation**	**US**
1	M	4 mo	13.7 (*N* 9–11)	4.9 (*N* 4–6.5)	6.2	262	0.8 (*N* <0.8)	Repeated vomiting, weight loss	NC (grade2)
2	F	10 mo	11.4 (*N* 9–11)	6.2 (*N* 4–6.5)	8.4	400	n/a	Asymptomatic	NC (grade 1)
3	M	9 mo	10.4 (*N* 9–11)	5.9 (*N* 4–6.5)	14.5	191	1 (*N* <0.6)	US done due to preauricular skin tag	NC (grade 2)–>NL (2y)
4	M	11 y	10.4 (*N* 8.8–10.8)	4.8 (*N* 3.3–5.4)	9.6	533	0.4 (*N* <0.2)	Abdominal pain	NL
5	M	1 mo	11.5 (*N* 9–11)	5.3 (*N* 4–6.5)	7.7	442	2 (*N* <0.8)	Asymptomatic	N
6	M	3 y	11.3 (*N* 8.8–10.8)	6.5 (*N* 3.2–5.8)	3.5	264	0.9 (*N* <0.4)	Renal colic	NL
7	M	7 y	9.8 (*N* 8.8–10.8)	5.6 (*N* 3.2–5.8)	5.5	202	0.4 (*N* <0.2)	Polyuria, nocturnal enuresis	NL
8	F	1 mo	11.1 (*N* 9–11)	7.8 (*N* 4–6.5)	7.5	364	1 (*N* <0.8)	Asymptomatic	N
9	F	7 mo	11 (*N* 9–11)	5.5 (*N* 4–6.5)	5.5	181	0.5 (*N* <0.6)	Urinary tract infection	NC (grade 3)
10	F	2 y	10.7 (*N* 8.8–10.8)	4.9 (*N* 4–6.5)	2	190	0.44 (*N* <0.4)	Abdominal pain	NC (grade 3)
Summary Data	6 males	Median 9.5 mo (range 1 mo−11 y)	Mean ± SD 11.1 ± 1	Mean ± SD 5.74 ± 0.87	Mean ± SD 7 ± 3.5	Mean ± SD 307 ± 130	Hypercalciuria-8, borderline calciuria-1		NC-5 NL-3 N-2

*F, female; M, male; Y, years; Mo, months; n/a, not available; NC, nephrocalcinosis; NL, nephrolithiasis; N, normal; Ca, calcium; PTH, parathyroid hormone; US, ultrasound*.

*Normal blood calcium and phosphor levels for age are noted in parenthesis (Tietz clinical guide to laboratory tests, 4th edition)*.

*Normal urinary calcium/creatinine ratios for age are noted in parenthesis (13, 14)*.

**Table 2:** Clinical and laboratory data at the last follow up visit.

**Patient *N***	**Age (y)/years of f/u**	**Treatment**	**Ca blood (mg/dl)**	**P blood (mg/dl)**	**PTH (pg/ml) (14–53)**	**1,25-D (pmol/l) (39–160)**	**Ca/Creatinine urine (mg/mg)**	**US**	**eGFR (mL/min/ 1.73 m^**2**^)**
1	5/4.6	Diet-Low sodium Low calcium Low vitamin D	10.1 (*N* 8.8–10.8)	5.8 (*N* 3.2–5.8)	5.5	130	0.6 (*N < * 0.4)	NC (grade 3)	120
2	2/1.1	No	9.1 (*N* 8.8–10.8)	4.4 (*N* 4–6.5)	17	210	N/a	N/a	159
3	8/7.1	TZD 12.5 mg/day Citrate Diet-Low sodium	9.6 (*N* 8.8–10.8)	5 (*N* 3.2–5.8)	13.6	191	0.28 (*N < * 0.2)	NL → stone excretion → N	140
4	17/6	TZD 25 mg/day Phosphor supplement Citrate	9.8 (*N* 8.4–10.2)	3.7 (*N* 2.5–5)	11.7	370	0.15 (*N < * 0.2)	N	159
5	2/1.9	No	10.1 (*N* 8.8–10.8)	5.2 (*N* 4–6.5)	18.8	170	0.5 (*N < * 0.4)	N	117
6	17/14	TZD 25 mg/day Citrate Diet- Low sodium	10.6 (*N* 8.4–10.2)	5.8 (*N* 2.5–5)	7	233	0.4 (*N < * 0.2)	NL	84
7	12/5	TZD 12.5 mg/day Citrate Diet- Low sodium	10.8 (*N* 8.4–10.2)	4.9 (*N* 3.2–5.8)	6	196	0.25 (*N < * 0.2)	NL	130
8	3/2.9	No	10.9 (*N* 8.8–10.8)	4.6 (*N* 3.2–5.8)	10.9	168	0.2 (*N < * 0.4)	N	161
9	2/1.4	Diet-Low sodium	10.3 (*N* 8.8–10.8)	4.4 (*N* 4–6.5)	14	262	0.6 (*N < * 0.4)	NC (grade 3)	192
10	4/2	TZD 6.25 mg/day Diet-Low sodium	11.2 (*N* 8.8–10.8)	4.9 (*N* 3.2–5.8)	14	158	0.46 (*N < * 0.4)	NC (grade 3)	88
Summary data	follow up (median) 3.75 (range 1.1–14 y)		Mean ± SD 10.3 ± 0.6	Mean ± SD 4.87 ± 0.6	Mean ± SD 12 ± 4.5	Mean ± SD 209 ± 65	Hypercalciuria-7	NC-3 NL-2 N-4	CKD-2

*eGFR, estimated glomerular filtration rate using modified Schwartz Formula (14); Y, years; n/a, not available; NC, nephrocalcinosis; NL, nephrolithiasis; N, normal; TZD, Disothiazide; Ca, calcium; PTH, parathyroid hormone; US, ultrasound*.

*Normal blood calcium values for age are noted in parenthesis*.

*Normal urinary calcium/creatinine ratios for age are noted in parenthesis (13, 14)*.

Mean blood calcium level at presentation was 11.1 ± 1 mg/dl. Four of our patients presented with hypercalcemia. In three of them this was revealed as an accidental finding, on a routine blood examination in the neonatal period (patients 5 and 8) or as a part of a work up of an unrelated medical problem (patient 2). The fourth patient presented in infancy with repeated vomiting and weight loss as a symptom of hypercalcemia (patient 1). Patients without hypercalcemia at presentation were also included to the cohort as they fulfilled other inclusion criteria. Mean PTH level at presentation was 7 ± 3.5 pg/ml (*N* 14–53 pg/ml). All patients presented with suppressed PTH except one who had PTH levels at low normal values in parallel to normal blood calcium levels, but suppressed PTH levels in follow up, hypercalciuria and nephrolithiasis, and was included into this cohort (patient 3). Mean 1,25-(OH)_2_D level at presentation was 307 ± 130 pmol/l (*N* 39–160 pmol/l). Eight patients had hypercalciuria and one had calcium excretion at the upper limit of normal values but had significant calciuria at follow up (the calcium creatinine ratio was 0.6 mg/mg at age 1.5 years). Five patients presented with nephrocalcinosis, which was revealed on renal ultrasound performed as part of investigation for abdominal pain (patients 4 and 10), urinary tract infection (patient 9), polyuria and nocturnal enuresis (patient 7), and skin tag (patient 3). Three patients presented with nephrolithiasis, one of them with renal colic (patient 6). Two patients had normal renal ultrasound at presentation.

### Genetic Analysis

Mutations in CYP24A1 were identified in three patients and a mutation in SLC34A3 was identified in one patient (Table 3).

**Table 3:** Genetic analysis.

**Patient *N***	**Gene**	**Mutation**
1	CYP24A1	Compound heterozygous: p.Arg396Trp (c.1186C>T)/p.Trp134Gly (c.400 T>G)
2	Not found	
3	Not found	
4	SLC34A3	Homozygous: p.Arg76Arg fs*60 (c.228delC)
5	Not found	
6	CYP24A1	Homozygous:p.E143del (c.427_429delGAA)
7	Not found	
8	Not found (SNIP in exon 13 homozygous)	
9	Not found (SNIP homozygous in exon 13 and 4)	
10	CYP24A1	Compound heterozygous: p.E143del (c.427_429delGAA)/p.Trp134Gly (c.400 T>G)
Total	CYP24A1 = 3	
	SLC34A3-1	

Patient 1 was found to be a compound heterozygote for two mutations in CYP24A1–p.Arg396Trp (c.1186C>T) and p.Trp134Gly (c.400 T>G). Patient 4 was found to be a homozygote for the p.Arg76Arg fs^*^60 (c.228delC) in SLC34A3. Patient 6 was found to be a homozygote for the p.E143del (c.427_429delGAA) in CYP24A1. Patient 10 was found to be a compound heterozygote for two mutations in CYP24A1 - p.E143del (c.427_429delGAA) and p.Trp134Gly (c.400 T>G) (Table 3).

### Treatment and Outcome

Treatment approaches and outcome parameters of each patient are summarized in Table 2. Treatment approaches included avoidance of sun exposure and vitamin D supplementation, sodium restriction and increased water intake, low calcium formula in one patient with symptomatic hypercalcemia (patient 1), citrate supplementation in patients with hypocitraturia, thiazide diuretics to reduce calciuria and phosphate supplementation in patients with SLC34A3 mutation.

**Figure 1:**
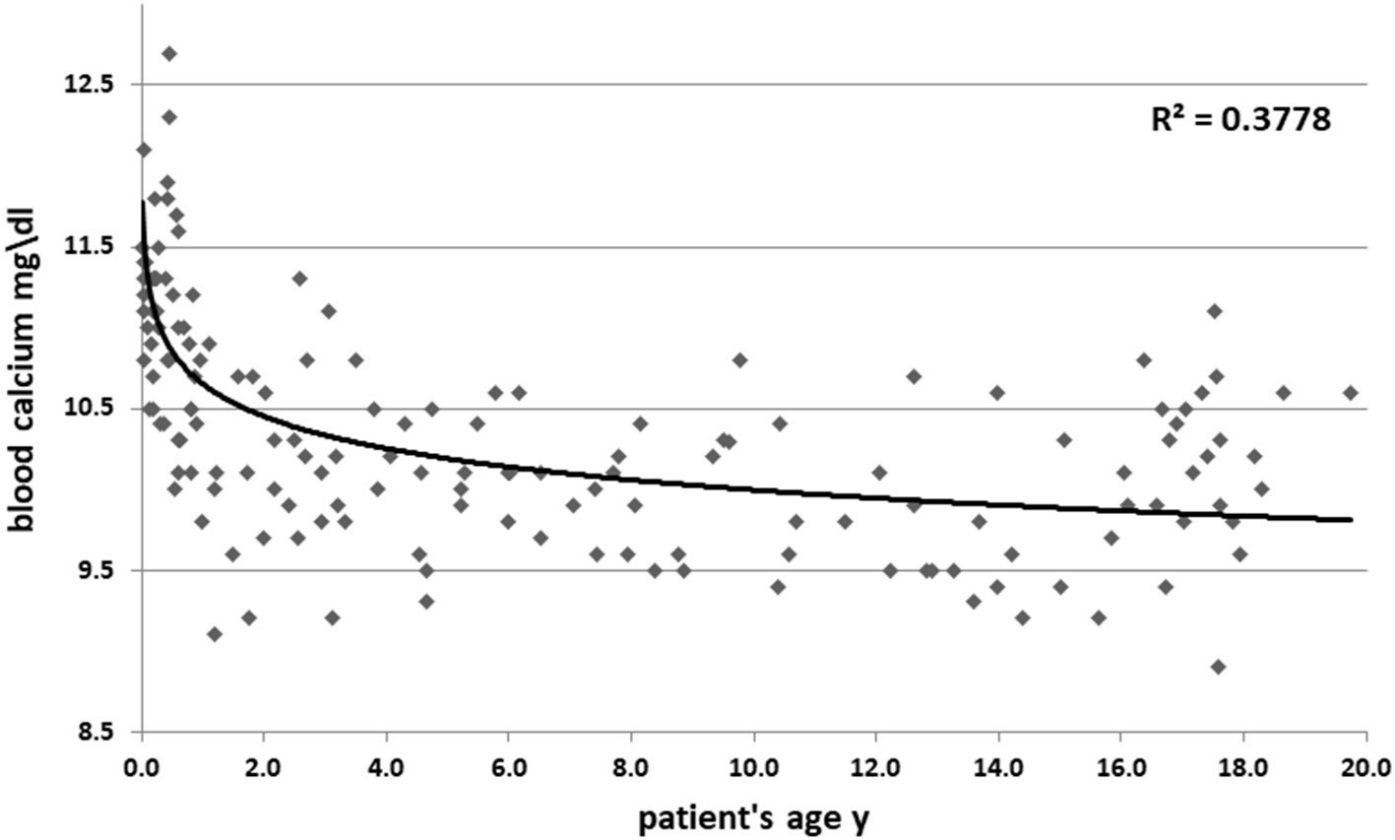
Blood calcium levels according to patient age. Y, years.

**Figure 2:**
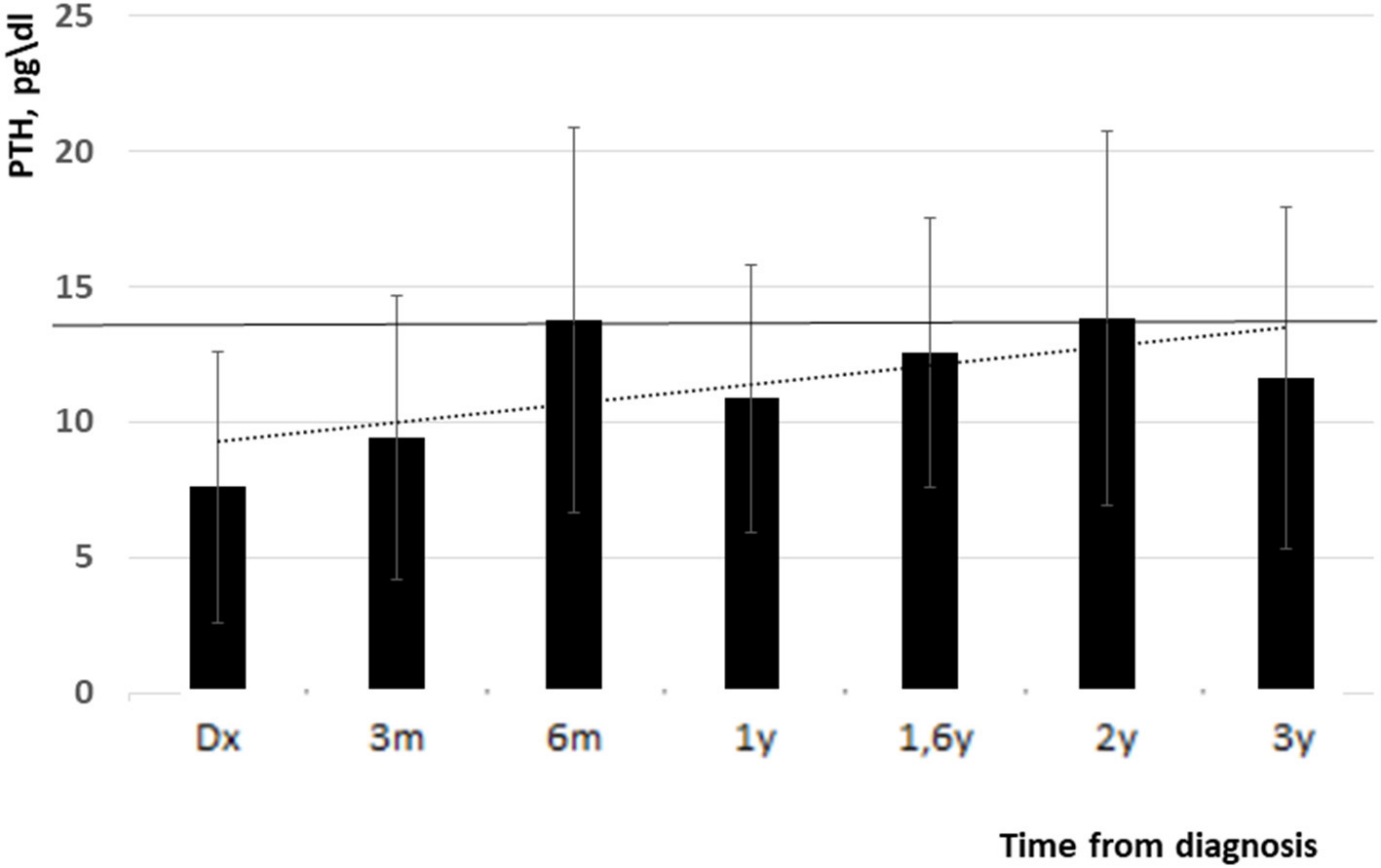
PTH levels from diagnosis and up to 3 years follow up (low normal limit is indicated with black line). Y, years, m, months.

High blood calcium decreased during follow up (11.05 ± 1 vs. 10.3 ± 0.6 mg/dl, *p* = 0.09). Figure 1 shows all the blood calcium levels that were recorded during the follow up. Decreasing blood calcium levels were reflected by increasing PTH (7 ± 3.5 vs. 12 ± 4.5 pg/ml, *p* = 0.01) (Figure 2).

**Figure 3:**
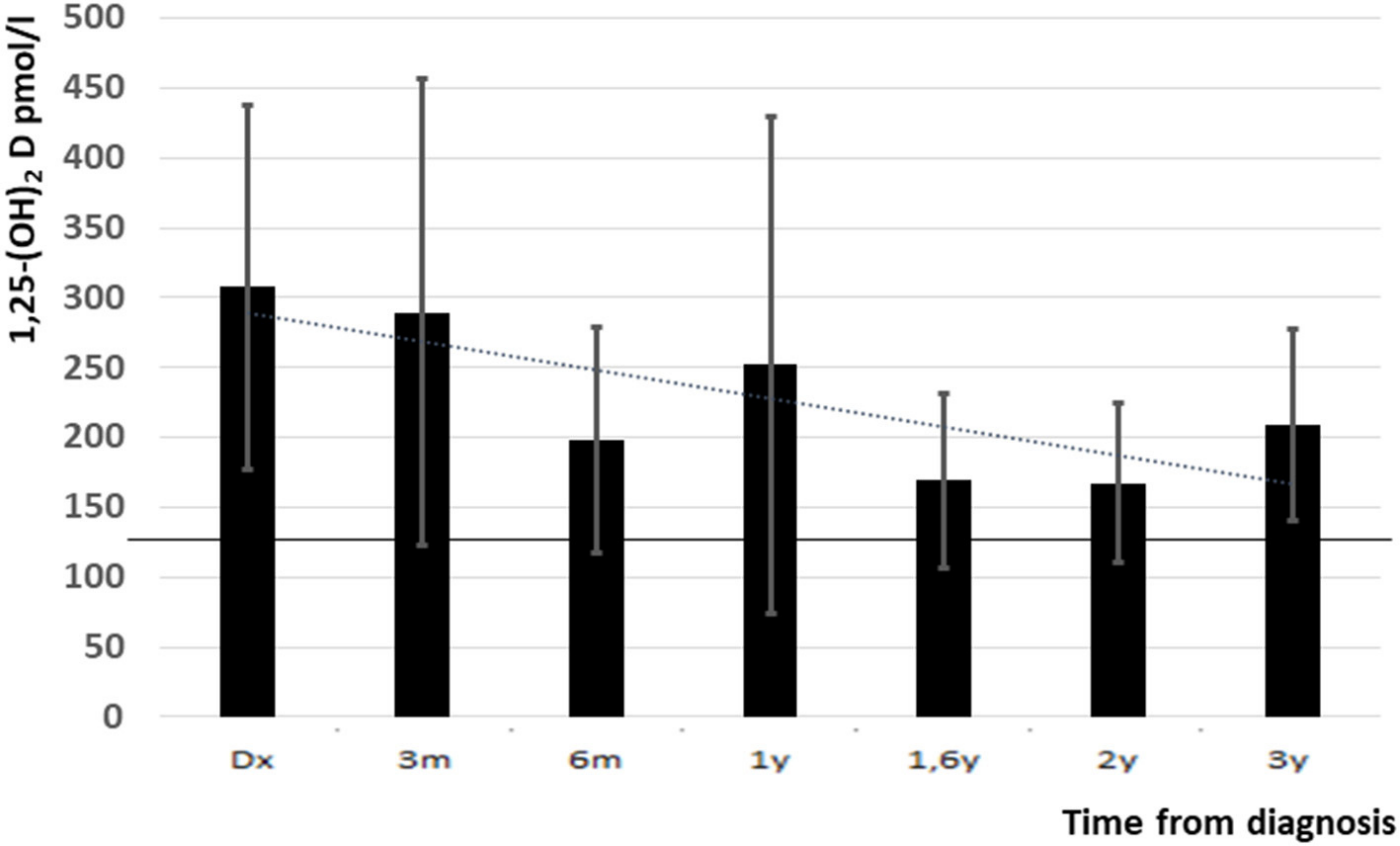
1,25-(OH)_2_D levels from diagnosis and up to 3 years follow up (upper normal level is indicated with black line). Y, years, m, months.

The elevated 1,25-(OH)_2_D level at presentation decreased during the study period but remained high at the last follow up visit (307 ± 130 vs. 209 ± 65 pmol/l, *p* = 0.05) (Figure 3). Mean levels of 25-(OH) D were slightly decreased during follow up (67 ± 44 nmol/l at presentation and 65 ± 26 nmol/l at the last follow up, normal range: 76–125 nmol/l).

Nine patients who underwent urine evaluation had hypercalciuria during the follow up period (Table 1). For one patient urine samples were not available. Hypercalciuria resolved spontaneously in one of the patients (patient 8) and with thiazide treatment in another one (patient 4). In the other 7 patients, hypercalciuria persisted, despite intervention in 6 of them. One patient with nephrocalcinosis was lost to follow up (patient 2). Patient 4, with nephrolithiasis at presentation and a SLC34A3 mutation, had a normal renal ultrasound at the last follow up, after treatment with a low salt diet, phosphate and citrate supplementation, and thiazide diuretics; urologic intervention was not required. Patient 3, with nephrocalcinosis grade 2, developed nephrolithiasis after 2 years follow up. However, with the abovementioned treatment, his hypercalciuria resolved, spontaneous stone excretion was observed, and his renal ultrasound became normal at the end of 8 years follow up. Three patients remained with nephrocalcinosis during the follow up (patients 1, 9, 10). These patients were treated with sodium restriction only (patients 1 and 9) and thiazide diuretics (patient 10). In one of them (patient 1), the grade of nephrocalcinosis increased from 2 to 3; for the other two, sonographic findings (grade 3 nephrocalcinosis) did not change during the follow up.

Patients 5 and 8 had normal ultrasound at presentation, and also during the follow up period. For two patients with nephrolithiasis at presentation (patients 6 and 7), the sonographic picture also did not change during the follow-up period.

Kidney function at the last follow-up was normal (estimated glomerular filtration rate (eGFR) above 90 ml/min/1.73 m^2^) in eight of the ten patients. Two patients had slightly reduced renal function (eGFR of 84–88 ml/min/1.73 m^2^) at the last follow up, both had CYP24A1 mutations and abnormal sonographic imaging (patients 6 and 10).

For patient 10, hypercalciuria and grade 3 nephrocalcinosis persisted, despite thiazide treatment. Her eGFR was 96 ml/min/1.73 m^2^ at first presentation, at age 2 years; and declined to 88 ml/min/1.73 m^2^ after 2 years follow up. Patient 6 presented at age 3 years with recurrent episodes of nephrolithiasis. Metabolic evaluation later in his life revealed hypercalciuria. Over the course of 12 years, his treatment consisted of a low sodium diet, thiazides, and citrate. His hypercalciuria improved but did not resolve, and recurrent episodes of nephrolithiasis persisted, with the need for recurrent urologic interventions. His renal function was normal for several years, but declined in the last 3 years of the follow up (his last eGFR was 84 ml/min/1.73 m^2^).

## Discussion

An increasing number of case reports and small case series have described patients with idiopathic hypercalcemia of infancy who carry bi-allelic CYP24A1 or SLC34A mutations (2, 3, 5–9). These patients presented with phenotypic and laboratory characteristics that were originally reported as idiopathic infantile hypercalcemia (IIH) (15–17), since the first described cohort of patients with CYP24A1 mutations in the 1950s. In addition, several publications have reported adolescent and adult patients with identical bi-allelic mutations in CYP24A1, who mainly presented with recurrent kidney stone disease or nephrocalcinosis, typically during childhood or adolescence (18–23). In those patients, laboratory examinations mostly demonstrated a milder degree of hypercalcemia, but suppressed PTH levels were similar to those in infants with IIH. In addition, a few case reports described women with CYP24A1 defects who developed hypercalcemia during pregnancy, which was associated with prematurity and intrauterine growth retardation (8, 24).

In contrast to the presumed good prognosis of a disease believed to be “transient” (12), data obtained in adult patients in recent years raise questions about the long-term prognosis and possible complications of sustained activation of vitamin D metabolism caused by defects in CYP24A1. In a number of patients with mutations in CYP24A1 or SLC34A, deterioration in renal function has been reported (3, 19, 21, 22, 25, 26). Only a few published reports have described the natural history of this condition, and the long-term prognosis is largely unknown (27). Ertl et al. (28) described 4 years of follow up of an infant with IIH and biallelic CYP24A1 mutations, and showed seasonal variations of vitamin D and calcium levels, with no effect on kidney function. Huang et al. (29) described the presentation, treatment, and outcomes of 11 children with IIH, with a mean follow up of 65 months. Hypercalcemia resolved in the majority of children by age 3 years. Nephrocalcinosis and persistent hypercalciuria were common. In a recent long-term follow-up of 18 patients with CYP24A1 and SLC34A1 mutations, impaired kidney function at a mean age of 23.8 years was found in 77%, including two adults with end-stage renal disease. Mild calciuria was observed in only one patient, but nephrocalcinosis of various degrees was found in 16 (30). These studies highlight the need for long-term follow up of patients with IIH.

We report long term follow up of a genetically heterogeneous, but phenotypically similar cohort. Our patients presented at different ages which probably can be explained by late diagnosis of asymptomatic condition. All the patients underwent genetic diagnosis but only in four of them mutations in CYP24A1 or SLC34A were found, which can be explained by existence of still unidentified mutations in genes regulating vitamin D metabolism. Despite this, we considered to describe all these patients as a cohort because of their common biochemical profile at presentation and during the follow up consistent with IIH. Six of the 10 patients presented in infancy, and the remaining four presented later in childhood. All our patients had hypercalciuria at presentation or during the early follow-up period. This is consistent with calcium metabolism disturbances described in this condition. In our cohort, high blood calcium and 1,25-(OH)_2_D at presentation decreased during follow up, in parallel to an increase in suppressed PTH. Interestingly, one patient with mutation in SLC3A3 gene coding for phosphate channel NapiIIc had normal phosphate levels at presentation which probably can be explained by effective activation of compensatory mechanism of FGF23 suppression due to phosphaturia, leading to increased 1,25-(OH)_2_D levels and absorptive hypercalciuria.

The best long treatment of patients with hypercalcemia, hypercalciuria, nephrocalcinosis, and nephrolithiasis due to CYP24A1 or SLC34A mutations is still unknown, but pathogenesis-based treatment consists of avoidance of vitamin D and sun exposure, hyperhydration, and a dietary recommendation of a low-sodium diet with normal calcium intake. Other long-term medical treatments that have been described include glucocorticoids, loop and thiazide diuretics, phosphate supplementation, proton pump inhibitors, and antifungals such as ketoconazole and fluconazole (31). Based on the pathogenesis, all our patients were advised to stop vitamin D supplementations and to avoid sun exposure. In one patient, who was diagnosed during infancy, formula feeding (containing 47 IU vitamin D/100 ml) was replaced with cow's milk without vitamin D supplement until a modified formula without any vitamin D content (Calcilo XD; Abbott Laboratories) could be provided (patient 1). A low sodium diet was recommended for all the patients with hypercalciuria, aimed to reduce urinary calcium excretion; six of them were compliant with this diet. Four patients received citrate supplementation due to low urine citrate levels (mean: 210 ± 48 mg/m^2^/1.73 m^2^, normal: above 365 mg/m^2^/1.73 m^2^) in the presence of hypercalciuria, as a measure to prevent calcium stone formation.

The addition of hydrochlorothiazide, a medication known to reduce renal calcium excretion due to increased reabsorption (32), can be considered to reduce hypercalciuria but may produce significant hypercalcemia. Thus, its use requires close follow-up of serum calcium levels in addition to calcium excretion follow up. Five of our patients were treated with thiazide diuretics due to hypercalciuria. Only one patient (patient 10), with a CYP24A1 mutation, had borderline hypercalcemia, which resolved after minimal dose reduction. No other side effects were noticed. This may be explained by slow and gradual dose increment. Lessening the degree of calciuria was shown in all the patients treated with thiazides, but hypercalciuria resolved in only one of them. Hypercalciuria resolved in one additional patient without any treatment. The variability in patients' response to treatment may be explained by the genetic heterogeneity of the cohort.

Patient 4 with mutation in SLC3A3 gene received phosphate supplementation, to diminish the effect of the phosphate loss on vitamin D metabolism.

Most of our cohort had abnormal sonographic findings during the follow up, including all the patients with identified mutations: five patients presented with nephrocalcinosis and three with nephrolithiasis. Two patients had normal renal ultrasound at presentation and during the follow up, despite overt hypercalciuria at the end of the follow up in one of them. Interestingly, no mutations were identified in these two patients. Overall, four patients had normal sonographic imaging at their last follow-up visit.

Eight of our patients had normal renal function during the entire follow-up period. Two patients developed grade 2 chronic kidney disease at the end of the follow-up, both of them had mutations in CYP24A1.

We were not able to show genotype-phenotype correlations in the severity of sonographic findings and renal function outcomes between the patients with and without mutations. However, slightly reduced renal function at the last follow up was observed only in the two patients with mutations in the CYP24A1 gene. In one of them, deterioration of renal function was observed only after several years of follow up, but the other one had reduced kidney function only 2 years after being diagnosed.

The main limitation of this study, is the relatively small cohort of patient with the rare condition described. One patient was lost to follow up. The data regarding the efficacy of the treatment is influenced by patient's compliance.

In summary, there is sparse data in the literature on long term follow up and renal function outcome of pediatric patients with infantile hypercalcemia. We report on long term follow up of pediatric cohort with described above clinical and biochemical profile. Over the study period, we found attenuation of the biochemical profile, including improvement in hypercalcemia, decreasing 1,25-(OH)_2_D levels, and increasing depressed PTH levels. On the other hand, in these patients, hypercalciuria and abnormal renal sonographic findings can persist despite treatment; and kidney function may deteriorate, determining unfavorable renal prognosis. In these patients genetic evaluation is recommended as well as long-term follow-up and treatment measures for preventing nephrocalcinosis and renal stone formation.

## Data Availability Statement

The original contributions presented in the study are included in the article/supplementary material, further inquiries can be directed to the corresponding author/s.

## Ethics Statement

The study was conducted according to protocol approved by the local committee on human research.

## Author Contributions

EG, SL, YB, HA, and MD followed up the patients, performed the data collection, and analysis. DD and LG performed the genetic analysis. EG and SL wrote the first draft of the manuscript and revised by MD. All authors contributed to the study conception and design and read and approved the final manuscript.

## Conflict of Interest

The authors declare that the research was conducted in the absence of any commercial or financial relationships that could be construed as a potential conflict of interest.

## Publisher's Note

All claims expressed in this article are solely those of the authors and do not necessarily represent those of their affiliated organizations, or those of the publisher, the editors and the reviewers. Any product that may be evaluated in this article, or claim that may be made by its manufacturer, is not guaranteed or endorsed by the publisher.
